# Absence of Polymorphisms in Codons 167, 198 and 200 of All Seven β-Tubulin Isotypes of Benzimidazole Susceptible and Resistant *Parascaris* spp. Specimens from Australia

**DOI:** 10.3390/pathogens11050490

**Published:** 2022-04-20

**Authors:** Murat Özben, Georg von Samson-Himmelstjerna, Malene K. B. Freiin von Streit, Edwina J. A. Wilkes, Kristopher J. Hughes, Jürgen Krücken

**Affiliations:** 1 Institute for Parasitology and Tropical Veterinary Medicine, Freie Universität Berlin, 14163 Berlin, Germany; murat_ozbn@hotmail.com (M.Ö.); samson.georg@fu-berlin.de (G.v.S.-H.); malene.v.streit@gmx.de (M.K.B.F.v.S.); 2 Department of Parasitology (Veterinary), Ankara University Graduate School of Health Sciences, 06230 Ankara, Turkey; 3 School of Agricultural, Environmental and Veterinary Sciences, Charles Sturt University, Wagga Wagga, NSW 2678, Australia; edwina.j.wilkes@gmail.com (E.J.A.W.); krhughes@csu.edu.au (K.J.H.)

**Keywords:** *Parascaris*, benzimidazoles, anthelmintic resistance, beta-tubulin paralogs, genotyping

## Abstract

Benzimidazoles resistance is widespread in strongyle parasitic nematodes and associated with polym orphisms in the codons 167, 198 and 200 of isotype 1 β-tubulin (tbb-1). In ascarids, benzimidazole (BZ) resistance has rarely been reported and in none of these cases were any of these polymorphisms detected. Here, available genome and transcriptome data from WormBase ParaSite were used to compare the complete β-tubulin reservoirs of *Parascaris univalens*, *Ascaris suum* and *Ascaris lumbricoides*. Adult *Parascaris* spp. specimens collected in Australia from horses after BZ treatment (susceptible, n = 13) or surviving BZ treatment and collected after ivermectin treatment (resistant, n = 10) were genotyped regarding codons 167, 198 and 200 using Sanger sequencing. Phylogenetic analyses clearly showed that there are no one-to-one ascarid orthologs of strongyle tbb-1 genes. In the reference genomes, as well as phenotypically susceptible and resistant *Parascaris* spp. from Australia, six out of seven β-tubulin genes showed a BZ-susceptible genotype (F167, E198, F200). The only exception were the testis-specific β-tubulin D genes from all three ascarid species that encode tyrosine at codon 200. This was observed independently of the BZ-susceptibility phenotype of *Parascaris* spp. These data suggest that different mechanisms lead to BZ resistance in ascarid and strongyle nematodes.

## 1. Introduction

*Parascaris* spp. are among the most pathogenic gastrointestinal nematodes of equines and are of particular importance regarding the health of foals and yearlings [1], where the prevalence of these parasites can be very high [2,3,4]. Pathology is induced by both migrating larvae in the liver and lungs and adults in the small intestine, where large numbers can lead to obstruction, colic and even rupture of the small intestine [1,3]. In horses, two species of the genus *Parascaris* are well known, i.e., *Parascaris univalens* and *Parascaris equorum*. Both species can only be reliably distinguished using karyotyping of early embryos in the two to four cell stage [5] or by isoelectric focussing of enzymes [6]. These methods cannot be conducted on frozen and fixed specimens and in most studies, identification of *Parascaris* species was not undertaken. Unfortunately, historically in many studies, samples were assigned to *P. equorum* or *P. univalens* without any experimental evidence. Although both species were regularly detected until the 1970s, there has been no publication of infections with *P. equorum* using karyotyping since 1989 [7,8]. However, few studies have used karyotyping for species identification since 1989, and it remains questionable whether the published data are representative.

Infections with *Parascaris* spp. are particularly problematic, since resistance against anthelmintics is widespread [9,10,11,12]. Resistance against macrocyclic lactones, in particular ivermectin, has been reported from Europe, North America, Asia and Australia [10,12]. Resistance to pyrantel [2,13] and benzimidazoles (BZ) [14,15,16] has also been reported occasionally. Regarding the pattern of resistance to drug classes, the situation in *Parascaris* spp. is different from strongyle nematodes, where BZ resistance is highly prevalent, pyrantel resistance occurs frequently and macrocyclic lactone resistance is rare [3,9,10,11,12]. This leads to a situation where foals and yearlings are frequently treated with multiple anthelmintic classes to decrease the disease burden caused by both parasite groups. However, multiple, presumably suboptimal treatments might further increase the speed of selection of drug-resistant parasite populations. Markers to diagnose parasite populations that can be expected to show resistance to particular drug classes would be extremely valuable to optimise treatment recommendations [17].

Although markers for BZ resistance are well established for many species of strongyle nematodes [17], the situation is remarkably different for ascarids. This is in part due to the fact that *Parascaris* spp. are the only ascarid species for which resistance has been frequently reported. For *Toxocara* spp., there is only a single report from 1994 stating low efficacy of treatment with pyrantel pamoate while three consecutive treatments of puppies with 50 mg/kg fenbendazole were 100% effective [18]. Recently, fenbendazole resistance in *Ascaridia dissimilis* in turkeys [19] and low efficacy of albendazole against *Ascaris suum* in school children in Rwanda [20] have been reported.

In strongyle nematodes, such as *Haemonchus contortus*, *Trichostrongylus colubriformis* and *Teladorsagia circumcincta*, BZ resistance is well known to be associated by single nucleotide polymorphisms (SNPs) in the isotype 1 β-tubulin gene [17]. Initially, the amino acid exchanges F200Y [21,22], F167Y [23,24] and sometimes E198A [25] were predominantly reported in many resistant parasite populations. Recently, additional exchanges in codons 198, in particular E198L and E198V in resistant strongyle nematodes, have raised attention at least in some geographical regions [26,27,28,29,30,31]. Based on the knowledge that these mutations can confer resistance to BZs, epidemiological surveys have been established to rapidly identify BZ-resistant parasite populations, using methods such as pyrosequencing [26,27,28,32,33,34,35,36,37,38,39], droplet digital PCR [31,40] or deep amplicon sequencing [29,41].

In ascarids, the situation is complicated by an apparently higher number of β-tubulin isotypes encoded in the genome [17] and the fact that no clear orthologs to the isotype 1 β-tubulin gene of strongyle nematodes have been identified so far. Moreover, in *A. lumbricoides*, presence of an F167Y polymorphism has been described, despite the fact that worms had a fully BZ-susceptible phenotype [42]. Krücken et al. [20] observed low efficacy of albendazole against *A. lumbricoides*, but were not able to identify any resistance associated polymorphisms in four β-tubulin isotypes. A recent study by Roose et al. [43] identified only susceptible genotypes expected to confer susceptibility to BZs at all three loci in six out of seven β-tubulin paralogs encoded in the *A. lumbricoides* and *A. suum* reference genomes. Only tbb-D encoded tyrosine (TAT) at codon 200, but the expression level of this isotype was found to be negligibly low and it was concluded that this cannot contribute to BZ resistance [43]. Deep amplicon sequencing for the two most widely and highly expressed *Ascaris* β-tubulin genes revealed no evidence for SNPs associated with BZ resistance [43]. The β-tubulin repertoire of *P. univalens* has recently been characterised by Martin et al. [16]. The authors identified a very closely related *P. univalens* ortholog to almost every *Ascaris* spp. Β-tubulin isotype. With the exception of the tbb-B’ gene, which was only identified in the *A. lumbricoides* and not the *A. suum* reference genome by Roose et al. [43], no *P. univalens* ortholog was detected [16]. Deep amplicon sequencing for all seven *P. univalens* β-tubulin paralogs confirmed the presence of tyrosine at codon 200 of the tbb-D gene but no polymorphisms in any of the other codons/isotypes. Malekpour et al. [16] also found no polymorphisms in bt-A sequences obtained from *Parascaris* spp. Collected in Iran.

The presence of multiple paralogs of β-tubulin suggests that these proteins have evolved to have different functions and presumably will show differences in expression level and expression pattern in different live cycle stages and tissues. For instance, the mec-7 and tbb-4 orthologs of *C. elegans* and *H. contortus* are expressed in only a few cell types and at much lower levels than other β-tubulin isoforms [44]. Thus, it is quite unlikely that polymorphisms in these genes can confer high levels of resistance, except if they have an essential function that cannot be replaced by any of the other orthologs, at least temporarily. Another aspect is that the genotypes of multiple isoforms presumably contribute to the phenotype simultaneously. In *C. elegans* for instance, there are three isoforms expressed at high levels in all tissues, i.e., tbb-1, tbb-2 and ben-1. Since tbb-1 and tbb-2 encode a tyrosine in codon 200, they are expected to be insensitive to the effects of BZs. Nevertheless, worms remain phenotypically susceptible until a loss of function mutation occurs in ben-1 [21,45] or ben-1 is rendered BZ-insensitive by one of the polymorphisms F167Y, E198E, E198L, E198V, E198I, E198T, E198K or F200Y [46,47]. Once ben-1 has been deleted or converted to a BZ-insensitive genotype, worms become phenotypically resistant although the isoforms tbb-4 and mec-7 can be assumed to be susceptible to the action of BZs since they encode phenylalanine at codons 167 and 200 and glutamate or serine at codon 198. The natural variation of BZ responses in *C. elegans* wild isolates is also dominated and explainable by high variation in the ben-1 locus [48]. In contrast, all β-tubulin isoforms encoded in the genome of susceptible *H. contortus* encode phenylalanine at codons 167 and 200 and glutamate in position 198. In this context, the F200Y exchange in the highly expressed isotype 1 β-tubulin gene is sufficient to confer resistance, which can be further increased by deletion of isotype 2 β-tubulin [22].

Although recent publications have characterised the β-tubulin genes of *A. suum*, *A. lumbricoides* [43] and *P. univalens* [16], none of these studies contained the complete β-tubulin repertoire of all three ascarid species in their phylogenetic analyses. In all three species, single orthologs of *C. elegans* mec-7 and tbb-4 genes were identified. In contrast, for the ben-1/tbb-1/tbb-2 group in *C. elegans* and *H. contortus*, there were no one-to-one ortholog relationships, and both studies identified separate groups of ben-1-like genes in Rhabditia (including Strongylida) and Ascarida suggesting that evolution of these β-tubulin genes by duplications and diversification occurred independently after separation of both groups. Unfortunately, the bootstrap values for these groups were only moderate to low, particularly for the group from Rhabditia (89% in Rosse et al. [43] and 0% in Martin et al. [16]). The first aim of the present study was to perform a phylogenetic analysis including the complete β-tubulin repertoire of the three ascarid species to further support the hypothesis that ben-1-like β-tubulin evolved independently in Rhabditia and Ascarida. In addition to data presented by Martin et al., we used available transcriptome data to compare the β-tubulin expression levels for all β-tubulin isoforms of the two very closely related species *A. suum* and *P. univalens* [49] and compared the genotypes at codons 167, 198 and 200 of the complete β-tubulin repertoire of *Parascaris* spp. specimens from one farm with BZ-susceptible worms and another farm with BZ-resistant worms.

## 2. Materials and Methods

### 2.1. Parasite Collection

Susceptible and resistant worms were collected on different farms. Susceptible *Parascaris* spp. were obtained from foals ≥ 12 weeks of age that had not received a previous anthelmintic treatment within 6 weeks of recruitment into the study. Faecal egg counts were determined using a modified McMaster method with a multiplication factor of 10 to calculate eggs per gram faeces (epg) from raw egg counts, as described previously [50]. The weight of foals was determined using weigh scales, and foals with ≥ 100 *Parascaris* epg were administered fenbendazole (Panacur 100, Coopers Animal Health, Macquarie Park, NSW, Australia) at a dose of 10 mg/kg by nasogastric intubation, by staff of the Veterinary Clinical Centre (Charles Sturt University, Wagga Wagga, NSW, Australia). Foals were kept in individual yards for 72 h and faeces were collected twice daily, and worms were collected from the faeces and stored in 70% ethanol. *Parascaris* worms resistant to fenbendazole were collected from another property with documented fenbendazole treatment failure on previous visits [15]. Recruited foals and weanlings were ≥ 12 weeks of age and had either not received a previous treatment or the last treatment was at least six weeks ago. After determining the body weight with weigh scales, foals with ≥ 100 *Parascaris* epg were administered 10 mg/kg fenbendazole (Panacur 100, Coopers Animal Health Macquarie Park NSW, Australia) by nasogastric intubation by staff of the Veterinary Clinical Centre. A second faecal egg count was obtained for all foals on days 14–17 post treatment to identify animals with persistent high egg counts, which were then treated with 0.2 mg/kg ivermectin per os (Equimec Paste, Boehringer Ingelheim, North Ryde NSW, Australia). Four foals (individual FECR between 81% and −270%) were kept in individual yards for 72 h and faeces were collected twice daily to obtain fenbendazole-resistant worms that were stored in 70% ethanol. Worms were shipped to Berlin for molecular analyses and stored at 4 °C until further use.

### 2.2. DNA Isolation

For DNA isolation, only the anterior part of a worm was used to prevent that male sperm or embryos contribute to the isolated DNA. Approximately 400 mg tissue was homogenised and DNA was isolated using the NucleoSpin Tissue Kit for genomic DNA (Macherey Nagel, Düren, Germany). DNA was quantified using a Take3 plate in an Epoch plate photometer (Biotek Instruments, Bad Friedrichshall, Germany) and DNA quality was checked using the A_260_/A_280_ absorption ratio. DNA was stored frozen at −20 °C until use for PCRs.

### 2.3. Identification of β-Tubulin Genes in the Parascaris univalens and Ascaris suum Genomes

In order to identify β-tubulin coding sequences, the predicted transcripts from the genomes of *P. univalens* (Bioproject number PRJNA386823) and *A. suum* (PRJNA62057) [51,52] were searched using the TBLASTN algorithm [53] and an isotype 1 β-tubulin protein sequence from *H. contortus* (GenBank accession number ABM92348.1) on the WormBase ParaSite server [54]. Hits were evaluated regarding query coverage and all transcripts encoding only partial β-tubulin proteins were excluded. Whenever less than 95% query coverage was obtained, a search for additional exons was conducted using the TBLASTN on the NCBI BLAST server. For this purpose, the genomic DNA was downloaded adding up to 6 kb flanking sequences before and after annotated exons. The same *H. contortus* β-tubulin protein sequence (ABM92348.1) was used as query in a TBLASTN search against these genomic sequences. Exons were identified manually as regions with high identity in the TBLASTN search. Exon/intron boundaries were identified around these high identity regions, following the rule that introns start with GT and end with AG. Subsequently, cDNA sequences were obtained by merging the exon sequences to a single cDNA.

### 2.4. Phylogenetic Analysis

The coding sequences of the complete repertoire of β-tubulin genes from *P. univalens*, *A. suum*, *C. elegans* (except of Celtbb-6, which is quite different from other nematode β-tubulins) and *H. contortus* were aligned codon-wise using MUSCLE as implemented in Mega 7.0.26 [55]. Alignments of cDNAs and proteins were exported as fasta files. DAMBE 7.0.35 [56] was used to estimate the degree of saturation separately for codon positions 1 and 2 and codon position 3 based on fully resolved sites in the cDNA alignment. For this purpose, the substitution saturation test [57,58] was applied. An alignment containing only codon positions 1 and 2 was exported and used in the following maximum-likelihood phylogenetic analysis. Poorly aligned regions were removed from each alignment using Gblocks 0.91b (server version) [59] applying all three options for less stringent selection (allow smaller blocks, allow gap positions within the final blocks, allow less stringent flanking positions). Protein, DNA and codons were chosen as sequence type for the protein, cDNA positions 1 and 2 and complete cDNA alignments, respectively. Three phylogenetic trees were calculated, one using the protein alignment, one only based on codon position 1 and 2 and one using the complete cDNA alignment applying codon-based substitution models. IQ-TREE 1.6.12 [60] was used on the IQ-TREE webserver [61]. In the same run, ModelFinder software [62] was used to identify the optimal substitution model, including models with rate heterogeneity over sites. The Bayesian Information Criterion was used to automatically select the optimal substitution model. Ultrafast bootstrapping [63] with 1000 pseudoreplicates with autocorrelation parameter set to 0.99, the Shimodaira-Hasegawa approximate likelihood ratio test (SH-aLRT) [64] and a Baysian approximation of the aLRT (aBayes) [65] were used to calculate node support values. The parameters’ perturbation strength and IQ-TREE stopping rule were used with their default values of 0.5 and 100, respectively. The resulting tree was visualised using FigTree v1.4.4 (Andrew Rambaut, https://github.com/rambaut/figtree/releases, accessed on 20 March 2022) and CorelDraw 20.1.0.708 (Corel Cooperation, Ottawa, ONT, Canada).

### 2.5. Comparison of Expression Levels between β-Tubulin Paralogs in Parascaris univalens and Ascaris suum

Expression data for *P. univalens* and *A. suum* were downloaded from WormBase ParaSite [55] from the GEO datasets SRP108385 and SRP005511, respectively [52]. These data contain information regarding expression from Illumina sequencing of RNA isolated from different live cycle stages of adult tissues. For *P. univalens*, data about male intestine, testis, male carcass (male organs after removal of intestine and testis: epidermis, muscles, neuronal system, excretion system), ovary and embryos were available. For *A. suum*, expression data for whole males, female reproductive system, ovary, testis, embryos and second stage larvae were obtained from the database. All data were given as transcripts per million (TPM). Since the number of replicates for the different live cycle stages and tissues ranged between one and six, only a mean value is presented, and no statistical comparisons were conducted.

### 2.6. PCRs and Sequencing

Amplification of genomic DNA fragments covering codons 167, 198 and 200 was performed with β-tubulin isotype-specific primer pairs that are given in Table S1. Reactions contained 0.4 M betaine, 1 µL genomic DNA, 200 µM dNTPs, 300 nM of each primer (Table S1) and 0.02 U/µL Phusion High Fidelity DNA polymerase (Thermo Fisher Scientific, Waltham, MA, USA) in 50 µL 1 × Phusion HF buffer. After an initial denaturation at 98 °C for 30 s, 40 cycles of denaturation at 98 °C for 10 s, annealing at a primer-specific temperature (Table S1) for 30 s and elongation at 72 °C for 30 s, reactions were finally incubated at 72 °C for 5 min. PCR reactions were analysed on 1.5% agarose gels and amplicons were purified from the reactions using the DNA Clean & Concentrator-5 kit (Zymo Research, Freiburg, Germany). For Sanger sequencing, purified PCR products were sent to LGC Genomics (Berlin, Germany). BioEdit 7.0.5.3 (Tom Hall, https://bioedit.software.informer.com/7.0/, accessed on 20 March 2022) software was used to visualise chromatograms that were manually inspected for the presence of SNPs in codons 167, 198 and 200.

## 3. Results

### 3.1. Nomenclature of Parascaris univalens and Ascaris suum β-Tubulin Paralogs

In the genomes of both *P. univalens* and *A. suum*, seven β-tubulin paralogs were identified (Table 1). As already pointed out by Roose et al. [43], the nomenclature of the β-tubulin isotypes is quite confusing and should be revised. However, the phylogenetic relationships between the different isotypes are very complex [16,43], and a nomenclature in accordance with suggested rules for naming parasitic nematode genes [66] is difficult. Therefore, we follow here the recently proposed nomenclature by Roose et al. [43], since this is clearly different from previously used names, although we are aware that this nomenclature is not in agreement with nomenclature rules. However, as long as there is no generally agreed set of gene names, this nomenclature is a very useful temporary solution. Table 1 summarises all different names used for the different isotypes and provides accession numbers. Tables S1 and S2 describe the complete β-tubulin repertoire, including exact location of the exons of *P. univalens* and *A. suum*, respectively.

Of the seven β-tubulin loci in the genome of *P. univalens* (PRJNA386823), six represented full-length β-tubulins, while the seventh (PgB04_g136_t01) was initially missing the 5′- and a small portion of the 3′-end (Table S2). By manual identification of exons, the 5′- but not the 3′-end could be obtained (PgB04_g136_t01me in Table S2). From PgR045_g070_t01 a small intron sequence of 60 bp was removed by manual editing (PgR045_g070_t01me in Table S2). From one of the sequences, (PgB10_g062_t01) 54 bp were removed from the 5′-prime end. This removed sequence did not contain any ATG start codon before the ATG that aligned to the start codon of the *H. contortus* isotype 1 β-tubulin used as query in the BLAST analyses.

Identical search strategies against the *A. suum* genome (PRJNA62057) identified only six genes encoding a β-tubulin gene (Table S3). Manual editing was required for two of these genes (AgR022_g106_t02, AgE31_g003_t01), for which 75 bp were removed that started with an ATG but only the third ATG in the gene model aligned to the start codon of isotype 1 β-tubulin from *H. contortus*, and therefore, the first part of the sequence was deleted. One of the predicted (AgE31_g003_t01) β-tubulin proteins was 546 amino acids long after manual editing, which suggests that the gene model is wrong in the 3′-region, but since the query coverage was 97% in the TBLASTN search, it can be assumed that it only contains additional sequences that cannot interfere with the phylogenetic analysis as it was conducted. The missing *A. suum* β-tubulin was identified using the *A. lumbricoides* ortholog as a query in a BLASTN search in GenBank. The identified entry comes from the same genome assembly, and thus, all seven β-tubulin isotypes were identified in the PRJNA62057 published by Wang et al. [52].

In contrast to *P. univalens* and *A. suum*, eight β-tubulin genes have been annotated in the *A. lumbricoides* genome (PRJEB4950). The additional isotype, named bt-B’ by Roose et al. [43], was neither annotated in the genomes of *A. suum* or *P. univalens*, nor did blast searches with the *A. lumbricoides* bt-B’ identify sequences in the reference genomes that could correspond to this isotype. Again, sequences of some isotypes were manually edited to remove additional sequences from the cDNA sequences as detailed in Table S3.

### 3.2. Phylogenetic Analysis of the β-Tubulin Repertoire of Ascaris sp. and Parascaris univalens

In order to obtain a phylogenetic tree of nematode β-tubulins, including the complete ascarid repertoire with improved branch support, it was aimed to take advantage of the additional variation information in the cDNAs compared to the protein alignment used previously. Therefore, cDNA sequences were aligned codon-wise. Initially, a test for substitution saturation was performed separately for codon position 3 and for codon positions 1 and 2. For codon position 3, the index for substitution saturation ISS for a symmetric tree was 0.71, which was slightly but not significantly higher (*p* = 0.333) than the critical ISSc threshold of 0.69, indicating that the third codon suffers from substitution saturation and these data are only poorly suitable for phylogenetic analyses. In contrast, for codon positions 1 and 2, ISS was significantly lower than ISSc (0.16 vs. 0.74; *p* < 0.001), suggesting that these sequences are suitable for phylogenetic analyses.

**Figure 1:**
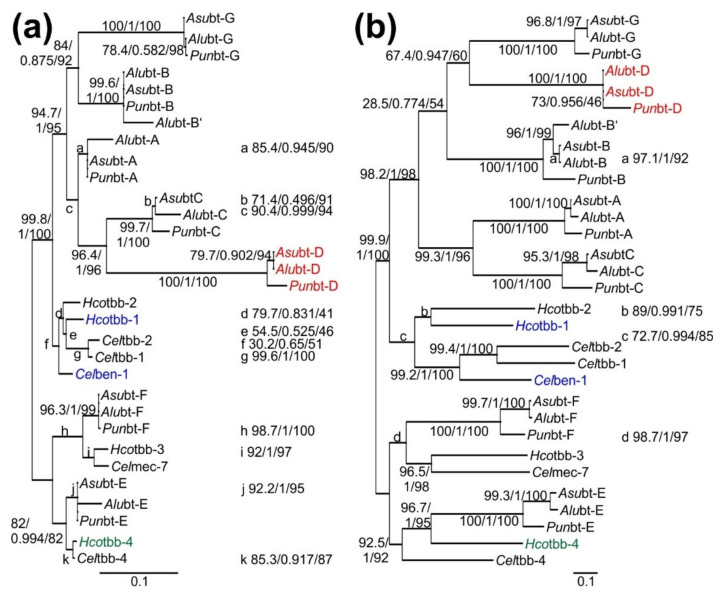
Phylogenetic trees of β-tubulin genes constructed by maximum likelihood analyses based on alignments of protein (**a**) and codon sequences (**b**). For the latter approach, a codon substitution model was applied. Coloured operational taxonomic units represent individual or groups of sequences that differ in their position between both trees and/or the tree based on codon-positions 1 and 2 of the cDNA sequence (Figure S1). The nomenclature of the genes follows the suggestion made by Roose et al. [43]. Branch support is given as SH-aLRT support (%)/aBayes support/ultrafast bootstrap support (%). For (A), several support values for grouping of very closely related ascarid β-tubulin genes are not shown. In all these cases, the SH-aLRT support was 0%. The scale bars indicate a distance of 0.1 substitutions per site.

Phylogenetic trees were constructed based on the protein sequences, the complete cDNA alignment and an alignment using only codon positions 1 and 2. The protein-based tree is shown in Figure 1a. The tree based on codon positions 1 and 2 of the cDNA alignment is very similar in the topology regarding the major groups and is provided in Figure S1. Since the substitution saturation at codon position 3 was quite high, it was decided to avoid construction of phylogenetic trees based on substitution models for position 3. Instead, the computationally more resource-demanding approach to construct a substitution model based on codons was evaluated. The resulting phylogenetic tree is shown in Figure 1b. Details about the substitution models for the three phylogenetic trees are provided in Tables S4–S6.

All three trees contain highly supported clusters for the Celtbb-4- and Celmec-7-like β-tubulins. The remaining β-tubulins fall into a single cluster, which is addressed as a ben-1-like β-tubulin cluster in the following. This cluster is clearly separated into a subcluster containing all ascarid members and a second containing all the ben-1-like β-tubulins from *C. elegans* and *H. contortus* (both Rhabditia). While the ascarid subcluster is highly supported in all three trees by all three statistical methods, support for the rhabditid ben-1-like cluster is poor in the protein-based tree and intermediate for the trees based on codon positions 1 and 2 and on codons (Figure 1, Figure S1). Within the rhabditid subcluster, the position of ben-1 varied between the different approaches. In the protein-based tree, it was located basal to all other members of the tree, which corresponds to its position in the previous analyses by Roose et al. [43] and Martin et al. [16]. In contrast, in the tree based on codon positions 1 and 2 only, ben-1 is placed in a sister position to *H. contortus* tbb-1 and tbb-2. Finally, in the tree based on a codon substitution model, ben-1 is located in a sister position to *C. elegans* tbb-1 and tbb-2. Only in the latter tree, the position of ben-1 has a high statistical support.

The position of *H. contortus* tbb-4 is obviously wrong in the codon-based tree, since it is placed with high support in a sister position to the ascarid tbb-4-like β-tubulins and not as a pair with *C. elegans* tbb-4. The latter pair of *C. elegans* and *H. contortus* tbb-4 is found in the protein-based tree and the tree calculated from codon positions 1 and 2 with moderate and high statistical support, respectively (Figure 1, Figure S1).

Finally, the position of the cluster of bt-D genes within the ascarid ben-1-like β-tubulins differs considerably between the different trees. All three trees suggest a close relationship between bt-B and bt-G and between bt-A and bt-C. For the tree obtained from the protein alignment, the bt-D group is placed next to bt-C, while both trees generated from cDNA sequences place bt-D sequences next to the bt-G cluster. In all cases, the support for the cluster containing bt-D, i.e., the cluster bt-A/bt-C/bt-D in Figure 1a or bt-B/bt-G/bt-D in Figure 1b and Figure S1 is only moderate, while the clusters containing only two groups are strongly supported.

Comparison of the ben-1-like clusters from ascarids and rhabditid nematodes furthers reveals considerably longer branch length for the ascarids although the latter are very closely related (same subfamily Ascaridinae) compared to *C. elegans* and *H. contortus* (same subclass Rhabditia). This suggests that all these clusters in ascarids represent ancient isotypes.

### 3.3. Expression Levels of β-Tubulin Paralogs in Parascaris univalens and Ascaris suum

**Figure 2:**
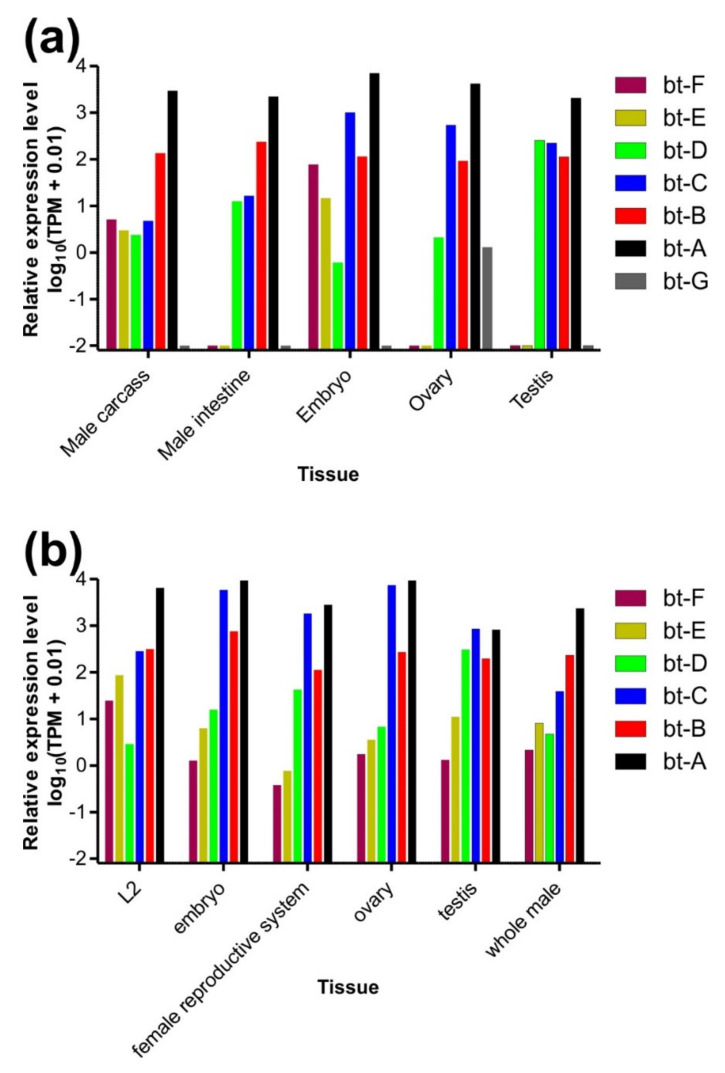
Tissue- and stage-specific expression data for *P. univalens* (**a**) and *A. suum* (**b**). Data were downloaded from WormBase ParaSite and represent the mean of 1–6 replicates. Data are provided as transcripts per million (TPM). In order to show TPM on a log scale, 0.01 was added to all values before plotting. Carcass, rest of the body after intestine and gonads have been removed. Embryo, five days after egg shedding with the faeces (32–64 cell stage). L2, second stage larvae.

Data of tissue-specific expression levels available from an RNASeq study [52] were available via WormBase ParaSite for each β-tubulin paralog in the *P univalens* genome. Since data are means of one to six replicates (depending on stage and tissue), no statistical analysis was conducted due to the too low number of replicates. Nevertheless, data clearly show that the isotype bt-A had by far the highest expression levels in terms of TPM and was the β-tubulin paralog with the highest expression level in all tissues (Figure 2a). The isoform with the second highest expression level in ovary and embryos was bt-C, whereas bt-B was the second in the male carcass and intestine. The isotype bt-D was the β-tubulin with the second highest expression level in the testis, while its level in all other tissues was very low (TPM ≤ 12.6). The isotype bt-G, the only isotype that was not annotated in the *A. suum* genome but only found using blast searches, was exclusively expressed in the ovary and even here only at very low levels (1.3 TPM) (Figure 2a).

In *A. suum*, β-tubulin isotypes show very similar tissue expression patterns as occurs in *P. univalens* (compare Figure 2a,b). The isotype bt-A is also the isotype with the highest expression level, except of the testis, where it is the second most abundant. Isotype bt-C is the most highly expressed in the testis and the second one in other samples except of L2, where bt-B was more frequently found, which is the third in ranking of expression levels in most other tissues. The isotype bt-D showed a similar testis-specific expression pattern as in *P. univalens*. For bt-G, which showed ovary-specific expression in *P. univalens*, no ortholog was annotated in *A. suum*, and therefore, no expression data were obtained from ParaSite WormBase.

### 3.4. Genotyping of Parascaris spp. Specimens from Australia

In the genome assemblies, almost all the β-tubulin isotypes encode phenylalanine in codon positions 167 and 200 and glutamate in codon position 198. The only exception was the bt-D isotype in *P. univalens*, *A. suum* and *A. lumbricoides*. This gene encodes tyrosine at codon position 200 in all three species. We, therefore, aimed to examine the situation in field samples comparing worms from BZ-resistant with worms from BZ-susceptible *Parascaris* spp. Populations. A total number of 13 *Parascaris* spp. Specimens were collected from the faeces of Australian horses after treatment with the BZ fenbendazole and 11 specimens were obtained after a first treatment with fenbendazole but only collected from faeces after a following second treatment with ivermectin. For ethanol-fixed specimens, discrimination of *P. univalens* and *P. equorum* is not possible using the only well-established standard, i.e., karyotyping of early division stages of eggs. In order to determine the species, intron sequences of β-tubulin genes were compared with those in the *P. univalens* reference genome. For all specimens, a >98% identity of sequences was observed for all PCR fragments including introns, suggesting that the specimens represent *P. univalens* and not *P. equorum*. However, in the absence of *P. equorum* reference sequences, this species cannot be finally excluded, and we refer to this material in the following as *Parascaris* spp.

In Table 2 and Table 3, the genotypes of codons 167, 198 and 200 are indicated for all seven β-tubulin isotypes of *P. univalens* in the Australian worms that were either susceptible to fenbendazole treatment and killed or BZ resistant and survived fenbendazole treatment. As for the reference genome assembly, all except of one gene showed completely susceptible genotypes (phenylalanine at codon positions 167 and 200 and glutamate at codon position 198). The only exception was bt-D that encoded tyrosine in codon position 200, an exchange that potentially leads to resistance. However, tyrosine was present in all specimens, regardless of whether worms survived a BZ treatment or not.

## 4. Discussion

Resistance of ascarid parasites to BZs has been reported rarely, but is expected to be of relevance for the treatment of horses and humans [14,15,18,20]. In horses, ascarid resistance to macrocyclic lactones is widespread [3,9,12] and resistance to both alternatives, BZs and pyrantel, has already been reported [2,13,14,15]. The BZs are the only drug class routinely used to deworm humans infected with soil-transmitted nematodes [67]. Depending on the prevalence of gastrointestinal nematodes, the WHO provides albendazole and mebendazole for treatment of preschool and school children once or twice a year in low-income countries [68]. While Krücken et al. [20] found low efficacy of albendazole against *A. lumbricoides* in school children in Rwanda, the authors were not able to identify molecular markers associated with BZ resistance. One possible explanation is that only four out of eight β-tubulin isotypes of *A. lumbricoides* were analysed.

With seven to eight β-tubulin paralogs [16,43], the repertoire of the ascarids is larger than that of the strongyle parasitic nematode *H. contortus*, which encodes four paralogs in its genome [44]. With one exception, closely related paralogs are found in all three ascarid species, *A. suum*, *A. lumbricoides* and *P. univalens*. The only exception, bt-B’ (ALUE_0001827701) was only found in the *A. lumbricoides* genome, but there was no hint of an ortholog in the reference genomes of *A. suum* and *P. univalens*. According to WormBase ParaSite, the genomes of *A. suum* and *P. univalens* show similar N50 length with 4.6 Mb and 1.8 Mb, respectively, while the value for *A. lumbricoides* is only 56 kb [54]. For the BUSCO statistics, describing content of essential single copy genes in the genomes, values for the *A. lumbricoides* assembly were lower (80.3% as single, 2.2% as double copy and 8.7% as fragmented, 91.2% in total) than for the assemblies of the *A. suum* (86.3% as single, 3.2% as double copy, 7.2% as fragmented, 96.7% in total) and *P. univalens* (88.4% as single, 2.6% as double copy, 5.8% as fragmented, 99.3% in total) genomes [55]. Thus, assembly quality cannot explain that a bt-B’ gene is only found in *A. lumbricoides*, since chances to obtain a complete set of β-tubulin genes should increase with genome assembly quality. Roose et al. [43] also did not find an ortholog in the second available genome assembly for *A. suum*. However, by deep amplicon sequencing of PCR products targeting bt-B, they found some hints that a second bt-B-like gene bt-B’ might also be present in *A. suum.*

The bt-D β-tubulin isotype was the only one in the genomes of *P. univalens*, *A. lumbricoides* and *A. suum* that showed a genotype suggesting no effects of BZs on its polymerisation kinetics. In the genome assemblies, all three genes show the presence of tyrosine in codon 200, and for *Parascaris* spp., this was confirmed for all specimens independently of their resistance status. All three genes show only low expression in all organs except in the testis, in which they are expressed at a similar level as the three isoforms that are most widely expressed (see Figure 2 and Roose et al. [43]). Due to its low expression levels, it is highly unlikely that in the presence of a BZ drug it will be able to functionally replace the three more widely and stronger expressed isoforms as previously concluded by Roose et al. [43]. However, a potentially possible resistance mechanism might be an increased level of expression tb-D in resistant worm populations. Transcriptome or RT-qPCR data to compare expression levels between BZ-susceptible and -resistant *P. univalens* populations would be required to evaluate this hypothesis.

In *A. lumbricoides*, the F167Y polymorphisms in the bt-A gene has previously been reported from children in Panama, Haiti and Kenya [42]. However, the high frequency of this genotype was not associated with any decrease in the efficacy of the BZ albendazole. The bt-A orthologs in *A. lumbricoides* [43], *A. suum* and *P. univalens* (Figure 2) show the highest expression levels among all β-tubulin isoforms in almost all tissues and stages tested. In this case, the presence of tyrosine in position 167 would be expected to have a considerable effect on drug responses as was observed for comparable mutations in *H. contortus* tbb-1 [17] and it remains unclear why this was not observed by Diawara et al. [42]. Possible explanations for this discrepancy are that tyrosine at codon position 200 does not have a substantial effect on polymerisation of ascarid tubulin dimers containing bt-A in the presence of a benzimidazole or that at least one of the other two β-tubulin isotypes in ascarids with relatively high expression level, i.e., bt-B and bt-C in ascarids, has an essential function that cannot be replaced by bt-A in the presence of a BZ.

With the *Parascaris* spp. Samples from Australia that were analysed in the present study, the situation is rather opposite. Despite apparent phenotypic resistance, there was no polymorphism correlated with resistance. This is in line with the observations reported by Martin et al. [16]. In principle, possible explanations are (i) polymorphisms in other positions in a β-tubulin gene, (ii) resistance mechanisms that are unrelated to changes in the drug target site and (iii) treatment failure due to factors including the overall health condition of the animal, drug application or quality, rather than genetically heritable resistance. Although for parasitic nematodes and *C. elegans*, codons in the three positions 167, 198 and 200 have been reported to correlate with resistance in strongyle nematodes, the situation is much more complex for other organisms such as fungi. Here, an additional three codons (positions 6, 50 and 240) and five genotypes (H6Y, Y50C, E198N, E198G and L240P) have been described, as reviewed by Ma et al. [69]. Thus, although up to now, only three codon positions have been involved in BZ resistance in strongyles, it cannot be excluded that selection at other positions leads to resistance in ascarids.

In addition to target site-related changes, for some nematodes, metabolism of BZs has been demonstrated. In *C. elegans*, it was shown that the cytochrome P450 Cyp-35d1 is able to oxidise thiabendazole, which is then conjugated to glucose and that downregulation of cyp-35d1 makes worms more susceptible to the effects of thiabendazole on egg laying in both a wild-type and a BZ-resistant, ben-1-deficient genetic background. Both *C. elegans* and *H. contortus* have been shown to metabolise several BZs (albendazole, mebendazole, thiabendazole, oxfendazole, fenbendazole and flubendazole) to hexose conjugates [70,71,72]. Data about metabolisation of anthelmintic drugs by ascarid nematodes have not been published, but changes in metabolic pathways, such as increased activity, obviously represent another option how anthelmintic resistance might be established in ascarids.

Drug application errors or insufficient quality of the drugs are additional reasons for low drug efficacy and treatment failure. The risk that efficacy data are obscured by such effects are particularly high if treatment is administered by untrained and/or unsupervised personnel. However, in the present study, trained veterinarians from the Veterinary Clinical Centre in Wagga Wagga treated the animals by nasogastric intubation of fenbendazole and the same personnel collected all samples. Moreover, BZ resistance was documented repeatedly on the farm from which the resistant parasites originated, but not on other farms [15]), which excludes poor health conditions of individual foals, poor drug quality and problems in drug application as explanations for low treatment efficacy.

In conclusion, comparison of phenotypically resistant and susceptible *Parascaris* spp. did not show any genetic alterations in the candidate codons that were expected to confer BZ resistance, although the complete β-tubulin repertoire was investigated. Since this is in accordance with recent data published by Martin et al. [16], other genetic changes should be considered that might be involved in BZ resistance of ascarids. This further contributes to the growing insight that BZ resistance mechanisms in strongyle and ascarid nematodes may differ substantially.

## Figures and Tables

**Table 1:** Overview of β-tubulin isotypes encoded in the reference genomes of *Parascaris univalens*, *Ascaris lumbricoides* and *Ascaris suum* and different nomenclatures.

Nomenclature Roose et al. [43]	Nomenclature Martin et al. [16]	Previous Names ^a^	Accession no. ^b^ *Parascaris univalens*	Accession no. ^b^ *Ascaris suum*	Accession no. ^b^ *Ascaris lumbricoidea*
bt-A	tbb-5	β-tubulin tbb-1 tbb-1.2 β-tubulin isotype 1	PgR007_g022_t01	AgB02_g235_t03	ALUE_0000927201
bt-B	tbb-8b	tbb-2	PgR003_g161_t01	AgR022_g106_t03	ALUE_0000986501
bt-B’	tbb-8a				ALUE_0001827701
bt-C	tbb-6	tbb-1	PgE153_g002_t01	AgE31_g003_t01	ALUE_0000494801
bt-D	tbb-7		PgB10_g062_t01	AgR043_g091_t01	ALUE_0001031701
bt-E	tbb-4	tbb-4	PgB004_g136_t01	AgB01_g252_t02	ALUE_0000949301
bt-F	tbb-3		PgB04_g135_t01	AgB01_g251_t03	ALUE_0000949201
bt-G	tbb-9		PgR045_g070_t01	CM024273: 10203269-10211838	ALUE_0001294101

^a^ For detailed references, see Roose et al. [43]. ^b^ All accession numbers refer to WormBase ParaSite, except for CM024273 (GenBank).

**Table 2:** Encoded amino acids at codon positions 167, 198 and 200 of the mec-7- and tbb-4-like β-tubulin paralogs in the genome of *Parascaris* spp.

β-Tubulin Paralog		PgB04_g135/bt-E			PgB04_g136/bt-F		
Sample ID	Res. status ^a^	Codon 167	Codon 198	Codon 200	Codon 167	Codon 198	Codon 200
S1	S	F	E	F	F	E	F
S2	S	F	E	F	F	E	F
S3	S	F	E	F	F	E	F
S4	S	F	E	F	F	E	F
S5	S	F	E	F	F	E	F
S6	S	F	E	F	F	E	F
S7	S	F	E	F	F	E	F
S8	S	F	E	F	F	E	F
S9	S	F	E	F	F	E	F
S10	S	F	E	F	F	E	F
S11	S	F	E	F	F	E	F
S12	S	F	E	F	F	E	F
S13	S	F	E	F	n.a.	n.a.	n.a.
R1	S	F	E	F	n.a.	n.a.	n.a.
R2	R	F	E	F	F	E	F
R3	R	F	E	F	F	E	F
R4	R	F	E	F	F	E	F
R5	R	F	E	F	F	E	F
R6	R	F	E	F	F	E	F
R7	R	F	E	F	F	E	F
R8	R	F	E	F	F	E	F
R9	R	F	E	F	n.a.	n.a.	n.a.
R11	R	F	E	F	F	E	F

^a^ Res. status, resistance status. Specimens that were collected after treatment with fenbendazole are labelled with S, while specimens that survived fenbendazole treatment collected after treatment with ivermectin are labelled with R. Susceptible and resistant worms originate from different farms. n.a., not available, i.e., no sequence data could be obtained.

**Table 3:** Encoded amino acids at codon positions 167, 198 and 200 of the ben-1-like β-tubulin paralogs in the genome of *Parascaris* spp.

β-Tubulin Paralog		PgR007_g022/bt-A			PgR003_g161/bt-B			PgE153_g002/bt-C			PgB10_g062/bt-D			PgR045_g070/bt-G		
Sample ID	Res. status^a^	Codon 167	Codon 198	Codon 200	Codon 167	Codon 198	Codon 200	Codon 167	Codon 198	Codon 200	Codon 167	Codon 198	Codon 200	Codon 167	Codon 198	Codon 200
S1	S	F	E	F	F	E	F	F	E	F	F	E	Y	F	E	F
S2	S	F	E	F	F	E	F	F	E	F	F	E	Y	F	E	F
S3	S	F	E	F	F	E	F	F	E	F	F	E	Y	F	E	F
S4	S	F	E	F	F	E	F	F	E	F	F	E	Y	F	E	F
S5	S	F	E	F	F	E	F	F	E	F	F	E	Y	F	E	F
S6	S	F	E	F	F	E	F	F	E	F	F	E	Y	F	E	F
S7	S	F	E	F	F	E	F	F	E	F	F	E	Y	F	E	F
S8	S	F	E	F	F	E	F	F	E	F	F	E	Y	F	E	F
S9	S	F	E	F	F	E	F	F	E	F	F	E	Y	F	E	F
S10	S	F	E	F	F	E	F	F	E	F	F	E	Y	F	E	F
S11	S	F	E	F	F	E	F	F	E	F	F	E	Y	F	E	F
S12	S	F	E	F	F	E	F	F	E	F	F	E	Y	F	E	F
S13	S	F	E	F	F	E	F	F	E	F	F	E	Y	F	E	F
R1	S	F	E	F	n.a.	n.a.	n.a.	F	E	F	F	E	Y	F	E	F
R2	R	F	E	F	F	E	F	F	E	F	F	E	Y	F	E	F
R3	R	F	E	F	F	E	F	F	E	F	F	E	Y	F	E	F
R4	R	F	E	F	F	E	F	F	E	F	F	E	Y	F	E	F
R5	R	F	E	F	F	E	F	F	E	F	F	E	Y	F	E	F
R6	R	F	E	F	F	E	F	F	E	F	F	E	Y	F	E	F
R7	R	F	E	F	F	E	F	F	E	F	F	E	Y	F	E	F
R8	R	F	E	F	F	E	F	F	E	F	F	E	Y	F	E	F
R9	R	F	E	F	F	E	F	F	E	F	F	E	Y	F	E	F
R11	R	F	E	F	F	E	F	F	E	F	F	E	Y	F	E	F

^a^ Res. status, resistance status. Specimens that were collected after treatment with fenbendazole are labelled with S, while specimens that survived fenbendazole treatment collected after treatment with ivermectin are labelled with R. Susceptible and resistant worms originate from different farms. n.a., not available, i.e., no sequence data could be obtained.

## Data Availability

Data are contained within the article or Supplementary Materials.

## Supplementary Materials

The following supporting information can be downloaded at: https://www.mdpi.com/article/10.3390/pathogens11050490/s1, Figure S1: Phylogenetic trees of β-tubulin genes constructed by maximum likelihood analyses based on an alignment of cDNA positions 1 and 2; Table S1: Transcripts in the *Parascaris univalens* genome (PRJNA386823) encoding proteins with high identity to isotype 1 β-tubulin from *Haemonchus contortus*; Table S2: Transcripts in the *Ascaris suum* genome (PRJNA62057) encoding proteins with high identity to isotype 1 β-tubulin from *Haemonchus contortus*; Table S3: Transcripts in the *Ascaris lumbricoides* genome (PRJEB4950) encoding proteins with high identity to isotype 1 β-tubulin from *Haemonchus contortus*; Table S4: Substitution model used for phylogenetic reconstruction based on protein sequences; Table S5: Substitution model used for codon-based phylogenetic reconstruction; Table S6: Substitution model for phylogenetic reconstruction based on codon positions 1 and 2.
