# Supplementary material for: Absence of Polymorphisms in Codons 167, 198 and 200 of All Seven β-Tubulin Isotypes of Benzimidazole Susceptible and Resistant Parascaris spp. Specimens from Australia
Source: Pathogens. 2022 Apr 20;11(5):490. doi: 10.3390/pathogens11050490 (PMC9143322; doi:10.3390/pathogens11050490)
Supplement: Supplementary file 1 [file pathogens-11-00490-s001.zip › pathogens-1673919-supplementary/Figure S1.pdf]

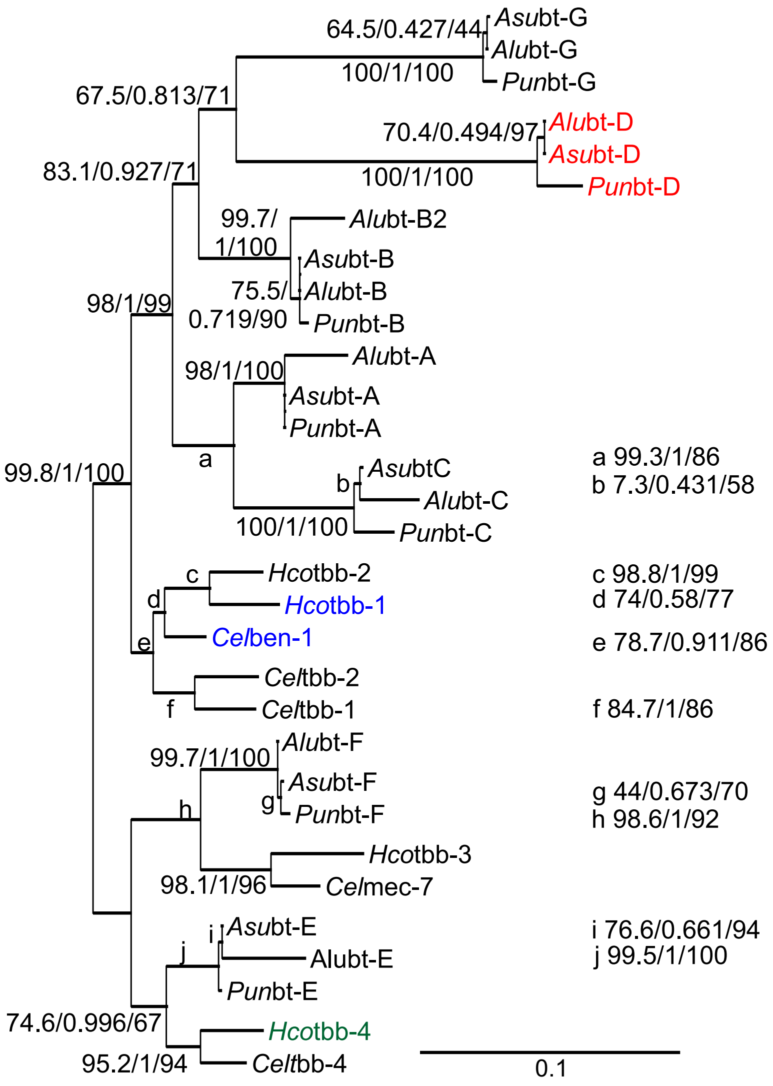

Figure S1. Phylogenetic trees of β-tubulin genes constructed by maximum likelihood analyses based on an alignment of cDNA positions 1 and 2. Coloured operational taxonomic units represent individual or groups of sequences that differ in their position between this tree and the trees presented in Figure 1. The nomenclature of the genes follows the suggestion made by Roose et al. [43]. Branch support is given as SH-aLRT support (%) / aBayes support / ultrafast bootstrap support (%). Several support values for grouping of very closely related ascarid β-tubulin genes are not shown. In all these cases, the SH-aLRT support was 0%. The scale bars indicate a distance of 0.1 substitutions per site.
