# Supplementary material for: Absence of Polymorphisms in Codons 167, 198 and 200 of All Seven β-Tubulin Isotypes of Benzimidazole Susceptible and Resistant Parascaris spp. Specimens from Australia
Source: Pathogens. 2022 Apr 20;11(5):490. doi: 10.3390/pathogens11050490 (PMC9143322; doi:10.3390/pathogens11050490)
Supplement: Supplementary file 1 [file pathogens-11-00490-s001.zip › pathogens-1673919-supplementary/Table S1.pdf]

Table S1. Transcripts in the *Parascaris univalens* genome (PRJNA386823) encoding proteins with high identity to isotype 1 β-tubulin from *Haemonchus contortus*

Transcript ID[a]	Nomenclature Roose et al. [43]	Nomenclature Martin et al. [16]	Annotated *C. elegans* ortholog[b]	Length (bp/aa[c])	Query coverage[d] (%)	Identity[d] (%)	Similarity[e] (%)	Location genome[e]	Location cDNA[e]
PgB04_g135_t01	bt-F	tbb-3	*mec-7*	1329/442	95	91.3	96.0	PgB04:1,953,671-1,964,238 (-)	
PgB04_g136_t01[f,g]	bt-E	tbb-4	*tbb-4*	916/305	68	92.1	97.7		
PgB04_g136_t01me[h]	bt-E	tbb-4	*tbb-4*	1278/426	95	91.5	96.9	PgB04:<br>1982336 - 1982392 (-)<br>1981706 - 1981814 (-)<br>1981151 - 1981261 (-)<br>1980417 - 1980532 (-)<br>1979909 - 1980037 (-)<br>1979599 - 1979714 (-)<br>1978892 - 1978987 (-)<br>1978310 - 1978409 (-)<br>1977262 - 1977448 (-)<br>1972810 - 1972928 (-)	 1 - 57<br>58 - 166<br>167 - 277<br>278 - 393<br>394 - 522<br>523 - 638<br>639 - 735<br>736 - 872<br>973 - 1159<br>1160 - 1278
PgB10_g062_t01	bt-D	tbb-7	n.a.	1356/451	99	79.8	91.3	PgB10:929520 - 937009 (+)	
PgE153_g002_t01	bt-C	tbb-6	n.a.	1353/450	100	85.7	94.0	PgE153:8,032 - 15,428 (+)	
PgR003_g161_t04	bt-B	tbb-8b	*tbb-6*	1368/455	97	90.8	96.6	PgR003:3157208 - 3178515 (+)	
PgR007_g022_t01	bt-A	tbb-5	n.a.	1353/450	98	93.2	97.7	PgR007:407010 - 413041 (+)	

PgR045_g070_t01	bt-G	tbb-9	*tbb-6*	1488/495	99	78.7	87.0	PgR045:1372050 - 1381671 (+)	
PgR045_g070_t01me[f]	bt-G	tbb-9	*tbb-6*	1428/475	99	82.1	90.8	PgR045:	
								1372050 - 1372106 (+)	1 - 57
								1373392 - 1373500 (+)	58 - 166
								1374127 - 1374353 (+)	167 - 393
								1375326 - 1375454 (+)	394 - 522
								1375747 - 1375909 (+)	523 - 685
								1377235 - 1377425 (+)	686 - 876
								1379551 - 1379646 (+)	877 - 972
								1379832 - 1380018 (+)	973 - 1159
								1381403 - 1381671 (+)	1160 - 1428

[a]For each locus, only the best matching transcript was included.

[b]Coding sequence including stop codon in base pairs/amino acids

[c]Using *Haemonchus contortus* isotype 1 β-tubulin as query in a TBLASTN search against the cDNA.

[d]Based on the BLOSUM62 similarity matrix.

[e]Details for individual exons are only provided if a different gene model than in the genome annotation was used.

[f]Version after manually editing the cDNA sequence.

[g]5'- and 3'-ends are missing.

[h]3'-end is missing.
