# Supplementary material for: Absence of Polymorphisms in Codons 167, 198 and 200 of All Seven β-Tubulin Isotypes of Benzimidazole Susceptible and Resistant Parascaris spp. Specimens from Australia
Source: Pathogens. 2022 Apr 20;11(5):490. doi: 10.3390/pathogens11050490 (PMC9143322; doi:10.3390/pathogens11050490)
Supplement: Supplementary file 1 [file pathogens-11-00490-s001.zip › pathogens-1673919-supplementary/Table S2.pdf]

Table S2. Transcripts in the *Ascaris suum* genome (PRJNA62057) encoding proteins with high identity to isotype 1 β-tubulin from *Haemonchus contortus*

Transcript ID[a]	Nomenclature Roose et al. [43]	Nomenclature Martin et al. [16]	Annotated *C. elegans* ortholog[b]	Length (bp/aa[c])	Query coverage[d] (%)	Identity[d] (%)	Similarity[e] (%)	Location genome[f]	Location cDNA[f]
AgB01_g251_t03	bt-F	tbb-3	*mec-7*	1329/443	95	91.6	96.3	AgB01: 3782705 – 3795903 (-)	
AgB01_g252_t02	bt-E	tbb-4	*tbb-4*	1335/444	97	90.8	96.6	AgB01: 3803075-3814443 (-)	
AgB02_g235_t03	*bt-A*	*tbb-5*	n.a.	1353/450	98	93.2	97.7	AgB02: 3973132-3980469 (+)	
AgE31_g003_t01	bt-C	tbb-6	n.a.	1941/646	100	86.4	94.6	AgE31:35111 - 46174 (+)	
AgE31_g003_t01me	bt-C	tbb-6	n.a.	1641/546	97	87.8	95.9	AgE31: 35111 - 35167 (+) 35988 - 36096 (+) 36896 - 37122 (+) 37774 - 37902 (+) 38297 - 38459 (+) 38927 - 39117 (+) 39342 – 39437 (+)	

								39640 - 39826 (+)	
								40660 - 40849 (+)	
								44486 - 44537 (+)	
								44937 - 45067 (+)	
								46064 - 46174 (+)	
AgR022_g106_t02	bt-B	tbb-8b	*tbb-6*	1443/480	97	90.8	96.6	AgR022: 1339279 - 1348771 (+)	
AgR022_g106_t02me[g]	bt-B	tbb-8b	*tbb-6*	1368/455	97	90.8	96.6	AgR022:	
								1341609 - 1341665 (+)	1 - 57
								1342505 - 1342613 (+)	58 - 166
								1343244 - 1343470 (+)	167 - 393
								1344927 - 1345055 (+)	394 - 522
								1345520 - 1345682 (+)	523 - 685
								1346298 - 1346488 (+)	686 - 876
								1346917 - 1347012 (+)	877 - 972
								1347305 - 1347491 (+)	973 - 1159
								1347982 - 1348771 (+)	1160 - 1368

AgR043_g091_t01	bt-D	tbb-7	n.a.	1356/452	99	80.9	91.3	AgR043: 1472802 - 1487383	
n.a.	bt-G	tbb-9	n.a.	1425/47485.5	99	82	91	CM024173.1:	
								10203269 - 10203325 (+)	1 - 57
								10204246 - 10204354 (+)	58 - 166
								10204989 - 10205216 (+)	167 - 394
								10206197 - 10206324 (+)	395 - 422
								10206637 - 10206799 (+)	423 - 585
								10207440 - 10207630 (+)	586 - 776
								10209713 - 10209808 (+)	777 - 872
								10209991 - 10210177 (+)	873 - 1059
								10211570 - 10211835 (+)	1060 - 1425

[a]For each locus, only the best matching transcript was included.

[b]Annotated *Caenorabditis elegans* ortholog in WormBase ParaSite

[c]Coding sequence including stop codon in base pairs/amino acids

[d]Using *Haemonchus contortus* isotype 1 β-tubulin as query in a TBLASTN search.

[e]Based on the BLOSUM62 similarity matrix.

[f]Details for individual exons are only provided if a different gene model than in the genome annotation was used.

[g]Version after manually editing the cDNA sequence.

n.a., not available
