# Supplementary material for: Absence of Polymorphisms in Codons 167, 198 and 200 of All Seven β-Tubulin Isotypes of Benzimidazole Susceptible and Resistant Parascaris spp. Specimens from Australia
Source: Pathogens. 2022 Apr 20;11(5):490. doi: 10.3390/pathogens11050490 (PMC9143322; doi:10.3390/pathogens11050490)
Supplement: Supplementary file 1 [file pathogens-11-00490-s001.zip › pathogens-1673919-supplementary/Table S3.pdf]

Table S3. Transcripts in the *Ascaris lumbricoides* genome (PRJEB4950) encoding proteins with high identity to isotype 1 β-tubulin from *Haemonchus contortus*

Transcript ID[a]	Nomenclature Roose et al. [43]	Nomenclature Martin et al. [16]	Annotated *C. elegans* ortholog[b]	Length (bp/aa[c])	Query coverage[d] (%)	Identity[d] (%)	Similarity[e] (%)	Location genome[e]	Location cDNA[e]
ALUE_0000927201-mRNA-1	bt-A	tbb-5	*ben-1*	1344/448	94	92.9	97.6	ALUE_scaffold0000470:57,079-61,212	
ALUE_0000986501-mRNA-1	bt-B	tbb-8b	*ben-1*	916/305	97	88.6	94.2	ALUE_scaffold0000555:88,104-95,361 (-)	
ALUE_0000986501-mRNA-1me[f]	bt-B	tbb-8b	*ben-1*	1365/455	97	90.1	96.6	ALUE_scaffold0000555:88,107-94241 (-)	
								94241 - 94185 (-)	1 - 57
								93345 - 93236 (-)	58-166
								92609 - 92283 (-)	167 - 393
								91048 - 90918 (-)	394 - 522
								90455 - 90293 (-)	523 - 685
								89996 - 89806 (-)	686 - 876
								89377 - 89282 (-)	877 - 972
								88989 - 88803 (-)	973 - 1159
								88312 - 88107 (-)	1160 - 1365
ALUE_0000494801-mRNA-1	bt-C	tbb-6	*ben-1*	1382/462	97	85.3	94.0	ALUE_scaffold0000077:278,876-284,803	
ALUE_0000494801-mRNA-1me[f]	bt-C	tbb-6	*ben-1*	1350/450	100	83.5	92.9	ALUE_scaffold0000077:278,876-284,800 (+)	
								278876 - 278920 (+)	1 – 45
								279928 - 280048 (+)	46 - 166
								280845 - 281072 (+)	167 - 393
								281721 - 281849 (+)	394 - 522
								282245 - 282407	523 - 685
								282875 - 283065 (+)	686 - 876

								283292 - 283387 (+)	877 - 972
								283590 - 283776 (+)	973 - 1159
								284610 - 284800 (+)	1160 - 1350
ALUE_0001031701-mRNA-1	bt-D	tbb-7	*ben-1*	1353/451	99	81.0	91.3	ALUE_scaffold0000629:12,771-18,776 (+)	
ALUE_0000949301-mRNA1	bt-E	tbb-4	*tbb-4*	1326/442	95	91.6	96.3	ALUE_scaffold0000501:88,208-98,499 (-)	
ALUE_0000949201-mRNA-1	bt-F	tbb-3	*mec-7*	1326/442	95	91.6	96.3	ALUE_scaffold0000501:63,518-77,036 (-)	
ALUE_0001294101-mRNA-1	bt-G	tbb-9	*ben-1*	1425/475	99	81.9	91.0	ALUE_scaffold0001173:40,528-50,229 (+)	

[a]For each locus, only the best matching transcript was included.

[b]Coding sequence including stop codon in base pairs/amino acids

[c]Using *Haemonchus contortus* isotype 1 β-tubulin as query in a TBLASTN search against the cDNA.

[d]Based on the BLOSUM62 similarity matrix.

[e]Details for individual exons are only provided if a different gene model than in the genome annotation was used.

[f]Version after manually editing the cDNA sequence.
