# Supplementary material for: Absence of Polymorphisms in Codons 167, 198 and 200 of All Seven β-Tubulin Isotypes of Benzimidazole Susceptible and Resistant Parascaris spp. Specimens from Australia
Source: Pathogens. 2022 Apr 20;11(5):490. doi: 10.3390/pathogens11050490 (PMC9143322; doi:10.3390/pathogens11050490)
Supplement: Supplementary file 1 [file pathogens-11-00490-s001.zip › pathogens-1673919-supplementary/Table S4.pdf]

Table S4. Substitution model used for phylogenetic reconstruction based on protein sequences

Model of substitution		LG+R3
Substitution rate category	Relative rate	Proportion
1	0.2110	0.7105
2	19.862	0.2390
3	74.250	0.0506
