# Supplementary material for: Absence of Polymorphisms in Codons 167, 198 and 200 of All Seven β-Tubulin Isotypes of Benzimidazole Susceptible and Resistant Parascaris spp. Specimens from Australia
Source: Pathogens. 2022 Apr 20;11(5):490. doi: 10.3390/pathogens11050490 (PMC9143322; doi:10.3390/pathogens11050490)
Supplement: Supplementary file 1 [file pathogens-11-00490-s001.zip › pathogens-1673919-supplementary/Table S5.pdf]

Table S5. Substitution model used for codon-based phylogenetic reconstruction

Model of substitution	KOSI07+F+G4		
State frequencies: (empirical counts from alignment)			
pi(AAA)=0.0143	pi(AAC)=0.0283	pi(AAG)=0.0178	pi(AAT)=0.0241
pi(ACA)=0.0141	pi(ACC)=0.0156	pi(ACG)=0.0146	pi(ACT)=0.0134
pi(AGA)=0.0106	pi(AGC)=0.0069	pi(AGG)=0.0027	pi(AGT)=0.0053
pi(ATA)=0.0045	pi(ATC)=0.0193	pi(ATG)=0.0442	pi(ATT)=0.0196
pi(CAA)=0.0227	pi(CAC)=0.0096	pi(CAG)=0.0257	pi(CAT)=0.0123
pi(CCA)=0.0182	pi(CCC)=0.0081	pi(CCG)=0.0086	pi(CCT)=0.0089
pi(CGA)=0.0106	pi(CGC)=0.0125	pi(CGG)=0.0029	pi(CGT)=0.0136
pi(CTA)=0.0066	pi(CTC)=0.0182	pi(CTG)=0.0111	pi(CTT)=0.0218
pi(GAA)=0.0392	pi(GAC)=0.0256	pi(GAG)=0.0350	pi(GAT)=0.0337
pi(GCA)=0.0190	pi(GCC)=0.0192	pi(GCG)=0.0109	pi(GCT)=0.0204
pi(GGA)=0.0300	pi(GGC)=0.0220	pi(GGG)=0.0049	pi(GGT)=0.0226
pi(GTA)=0.0098	pi(GTC)=0.0175	pi(GTG)=0.0208	pi(GTT)=0.0205
pi(TAC)=0.0186	pi(TAT)=0.0166	pi(TCA)=0.0119	pi(TCC)=0.0114
pi(TCG)=0.0133	pi(TCT)=0.0100	pi(TGC)=0.0104	pi(TGG)=0.0092
pi(TGT)=0.0090	pi(TTA)=0.0044	pi(TTC)=0.0356	pi(TTG)=0.0151
pi(TTT)=0.0168			
Model of rate heterogeneity: Gamma with 4 categories			
Gamma shape alpha:	509.515		
Category	Relative_rate	Proportion	
1	0.8280	0.2500	
2	0.9491	0.2500	
3	10.397	0.2500	
4	11.832	0.2500	

Relative rates are computed as MEAN of the portion of the Gamma distribution falling in the category.
