# Supplementary material for: Absence of Polymorphisms in Codons 167, 198 and 200 of All Seven β-Tubulin Isotypes of Benzimidazole Susceptible and Resistant Parascaris spp. Specimens from Australia
Source: Pathogens. 2022 Apr 20;11(5):490. doi: 10.3390/pathogens11050490 (PMC9143322; doi:10.3390/pathogens11050490)
Supplement: Supplementary file 1 [file pathogens-11-00490-s001.zip › pathogens-1673919-supplementary/Table S6.pdf]

Table S6. Substitution model for phylogenetic reconstruction based on codon positions 1 and 2.

Model of substitution			TIM2e+G4		
Rate parameter		R:			
A-C:		16.512			
A-G:		20.759			
A-T:		16.512			
C-G:		10.000			
C-T:		26.170			
G-T:		10.000			
State frequencies: (equal frequencies)					
Rate matrix Q					
	A	-1.076	0.3304	0.4154	0.3304
	C	0.3304	-1.054	0.2001	0.5236
	G	0.4154	0.2001	-0.8156	0.2001
	T	0.3304	0.5236	0.2001	-1.054
Model of rate heterogeneity: Gamma with 4 categories					
Gamma shape alpha:			0.2535		
Category	Relative rate	Proportion			
1	0.002281	0.25			
2	0.06927	0.25			
3	0.5089	0.25			
4	3.42	0.25			

Relative rates are computed as MEAN of the portion of the Gamma distribution falling in the category.
